# Personalized Treatment Strategies via Integration of Gene Expression Biomarkers in Molecular Profiling of Laryngeal Cancer

**DOI:** 10.3390/jpm14101048

**Published:** 2024-10-10

**Authors:** Antonino Maniaci, Giovanni Giurdanella, Carlos Chiesa Estomba, Simone Mauramati, Andy Bertolin, Marco Lionello, Miguel Mayo-Yanez, Paolo Boscolo Rizzo, Jerome R. Lechien, Mario Lentini

**Affiliations:** 1 Department of Medicine and Surgery, University of Enna “Kore”, 94100 Enna, Italy; giovanni.giurdanella@unikore.it (G.G.); marlentini@tiscali.it (M.L.); 2 ASP Ragusa-Hospital Giovanni Paolo II, 97100 Ragusa, Italy; 3 Head and Neck Study Group, Young Otolaryngologists-International Federation of Otorhinolaryngological Societies, 13005 Paris, France; chiesaestomba86@gmail.com (C.C.E.); miguelmmy@gmail.com (M.M.-Y.); jerome.lechien@umons.ac.be (J.R.L.); 4 Department of Otorhinolaryngology-Head and Neck Surgery, Hospital Universitario Donostia, 20003 San Sebastian, Spain; 5 Department of Otolaryngology Head Neck Surgery, University of Pavia, IRCCS Policlinico San Matteo Foundation, 27100 Pavia, Italy; simone.mauramati@gmail.com; 6 Department Otorhinolaryngology, Vittorio Veneto Hospital (ML, AB), Anesthesia and Intensive Care, Vittorio Veneto Hospital, 31029 Vittorio Veneto, Italy; andy.bertolin@aulss2.veneto.it (A.B.); marco.lionello@aulss2.veneto.it (M.L.); 7 Department of Otorhinolaryngology-Head and Neck Surgery, Hospital San Rafael (HSR), 15006 A Coruña, Spain; 8 Department of Medical, Surgical and Health Sciences, Section of Otolaryngology, University of Trieste, 34127 Trieste, Italy; paolo.boscolorizzo@units.it; 9 Department of Otorhinolaryngology and Head and Neck Surgery, CHU de Bruxelles, CHU Saint-Pierre, School of Medicine, 64000 Brussels, Belgium

**Keywords:** laryngeal cancer, gene expression profiling, molecular biomarkers, personalized medicine, oncogenomics

## Abstract

Laryngeal cancer poses a substantial challenge in head and neck oncology, and there is a growing focus on customized medicine techniques. The present state of gene expression indicators in laryngeal cancer and their potential to inform tailored therapy choices are thoroughly examined in this review. We examine significant molecular changes, such as TP53, CDKN2A, PIK3CA, and NOTCH1 mutations, which have been identified as important participants in the development of laryngeal cancer. The study investigates the predictive and prognostic significance of these genetic markers in addition to the function of epigenetic changes such as the methylation of the MGMT promoter. We also go over the importance of cancer stem cell-related gene expression patterns, specifically CD44 and ALDH1A1 expression, in therapy resistance and disease progression. The review focuses on indicators, including PD-L1, CTLA-4, and tumor mutational burden (TMB) in predicting immunotherapy responses, highlighting recent developments in our understanding of the intricate interactions between tumor genetics and the immune milieu. We also investigate the potential for improving prognosis accuracy and treatment selection by the integration of multi-gene expression panels with clinicopathological variables. The necessity for uniform testing and interpretation techniques is one of the difficulties, in implementing these molecular insights into clinical practice, that are discussed. This review seeks to provide a comprehensive framework for promoting personalized cancer therapy by combining the most recent data on gene expression profiling in laryngeal cancer. Molecularly guided treatment options may enhance patient outcomes.

## 1. Introduction

A large percentage of head and neck cancers are laryngeal cancers, which are a major worldwide health concern. It is the most prevalent malignancy of the upper aerodigestive tract and makes up about 1% to 2% of all cancer cases globally [1]. Squamous cell carcinoma is the most common type of cancer in the larynx, accounting for over 90% of the cases. The larynx is a complicated organ that is necessary for breathing, swallowing, and producing voice [2]. Geographically, laryngeal cancer incidence varies globally; higher rates are seen in areas with higher rates of alcohol and tobacco use, which is consistent with the substantial correlation between these risk factors and the development of the illness [3]. The etiology of laryngeal cancer is complicated, resulting from the interaction of genetic predisposition, lifestyle decisions, and environmental variables. Although alcohol and tobacco use continue to be the main risk factors, accounting for around 75% of cases [4,5], other factors have also been linked to the pathogenesis of laryngeal cancer, including dietary habits, occupational exposures, and human papillomavirus (HPV) infection [6,7]. The clinical presentation and prognosis of laryngeal tumors are greatly influenced by their anatomical position, which can be classed as supraglottic, glottic, or subglottic. Glottic cancers typically have a better prognosis since they typically appear with symptoms sooner [8]. Over the past few decades, there has been no discernible improvement in the overall survival rates for laryngeal cancer despite advancements in diagnostic methods and treatment modalities [9]. The majority of current treatment plans include radiation, chemotherapy, and surgery in a multimodal manner, which frequently results in severe morbidity and a reduced quality of life [10]. Because laryngeal cancer varies widely in terms of its molecular makeup and clinical behavior, more individualized treatment plans are required in order to maximize therapeutic benefit and reduce side effects. Over the past 20 years, customized medicine has become increasingly popular in oncology, transforming cancer diagnosis, prognosis, and treatment [11]. This paradigm shift toward customized therapeutic approaches is motivated by the realization that every cancer is different from the others molecularly, which requires customized techniques for the best care. Personalized medicine presents potential benefits for laryngeal cancer patients, including enhanced treatment selection, more precise risk classification, and the creation of targeted medicines based on the unique molecular profile of each tumor [12]. It is impossible to overestimate the significance of tailored medicine in oncology, especially in cases of laryngeal cancer. Clinicians may be able to anticipate therapy responses, identify patients at high risk of recurrence, and customize interventions to improve efficacy while reducing side effects by combining molecular biomarkers, genetic analysis, and unique patient characteristics [13]. This strategy can increase survival rates, but it can also improve quality of life by sparing patients who are unlikely to benefit from needless interventions. Novel treatment targets and possible biomarkers for laryngeal cancer have been identified more rapidly due to recent developments in high-throughput genomic technologies [14]. Specifically, gene expression profiling has become a formidable tool for comprehending the molecular basis of treatment resistance, metastatic potential, and tumor behavior [15,16,17]. Researchers can uncover molecular fingerprints linked to different clinicopathological characteristics and treatment outcomes by simultaneously studying the expression patterns of thousands of genes. This allows for more accurate prognostication and treatment selection [18]. The conversion of molecular discoveries into clinical practice is still difficult despite these encouraging advancements. Before being used in normal clinical decision-making, prospective biomarkers must be carefully validated and interpreted due to the complexity of cancer biology, tumor heterogeneity, and the dynamic nature of gene expression patterns [19]. However, widespread adoption is severely hampered by the expense and technical know-how needed for thorough molecular profiling, especially in environments with limited resources [20]. A thorough examination of the state of gene expression biomarkers today and their prospective uses in personalized treatment is necessary, given the quickly changing field of molecular oncology and the urgent need for better management approaches in laryngeal cancer. By combining the most recent findings on gene expression profiling in laryngeal cancer and its consequences for individualized treatment strategies, this scoping review seeks to fill this gap in the literature. This scoping review has three main goals in mind. We aimed to give a thorough summary of the state of gene expression biomarkers in laryngeal cancer at the moment, taking into account both their predictive and prognostic significance. We also investigated how these biomarkers might be used to tailor treatment plans, including how they might be used for risk assessment, treatment choice, and the creation of targeted medicines. At least, we pointed out the areas of laryngeal cancer personalized medicine that show promise for further investigation and to fill in knowledge gaps. By focusing on these goals, this review hopes to advance our understanding of molecular profiling in laryngeal cancer and make it easier to apply what we learn to better clinical outcomes. With a focus on precise and tailored techniques, this review seeks to critically analyze the potential of gene expression indicators to transform the treatment of laryngeal cancer patients at a time when a new era in cancer management is about to dawn.

## 2. Materials and Methods

The PRISMA-ScR (Preferred Reporting Items for Systematic Reviews and Meta-Analyses Extension for Scoping Reviews) criteria were followed in the conduct of this scoping study. The research question was identified, a literature search was conducted, studies were chosen, data were charted, and the results were synthesized.

### Literature Search and Research Question

“What is the current state of gene expression biomarkers in laryngeal cancer, and how can they be integrated into personalized treatment strategies?” was the main research question that served as the basis for this review. We searched all electronic databases, including PubMed, Embase, Web of Science, and the Cochrane Library, in great detail. The search approach comprised MeSH term combinations and keywords associated with the following terms: “molecular profiling”, “biomarkers”, “laryngeal cancer”, “gene expression”, and “personalized medicine”. Only English-language articles released between January 2000 and April 2024 were included in the search. The abstracts and titles of the retrieved publications were vetted by two separate reviewers. The following criteria were satisfied by the included studies: (1) They had to do with laryngeal cancer; (2) They had to look at gene expression biomarkers; (3) They had to address the implications for individualized treatment. The eligibility of the full-text articles was then determined. A third reviewer was consulted or discussed with in order to settle disagreements. To chart pertinent data from included research, a consistent data extraction form was utilized. The following information was retrieved from the data: author(s), publication year, study design, sample size, biomarkers examined, gene expression analysis techniques, major conclusions, and implications for tailored medicine. A narrative synthesis approach was chosen due to the heterogeneity of the research and the scope nature of this review. Themes such as the genetic landscape, epigenetic modifications, prognostic biomarkers, predictive biomarkers, and difficulties in clinical implementation were used to group the findings. We conferred with specialists in the fields of molecular pathology and head and neck oncology to strengthen the validity of our findings. Their observations were useful in understanding the findings and suggesting areas for further investigation. This approach made it possible to conduct a thorough and methodical investigation of the state of gene expression biomarkers in laryngeal cancer, which laid a strong basis for talking about their potential in individualized treatment plans.

## 3. Genetic Landscape of Laryngeal Cancer

**Figure 1:**
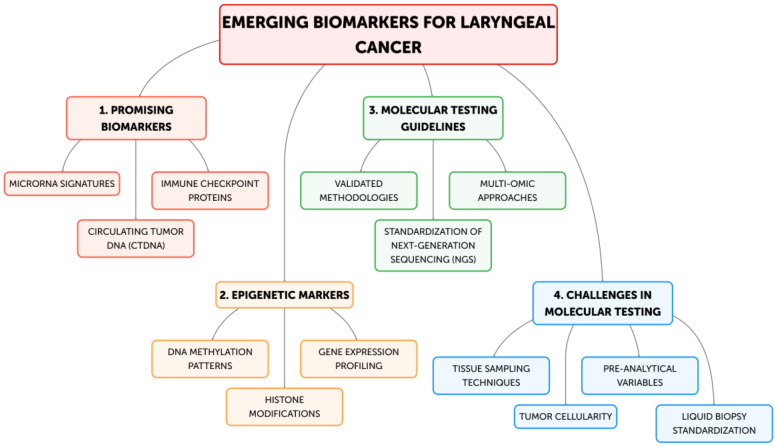
New emerging biomarkers and the need for standardization of molecular testing in laryngeal cancer.

Laryngeal cancer’s genetic landscape is defined by a wide range of molecular changes that affect the disease’s onset, course, and response to treatment. It is essential to comprehend these genetic alterations in order to create tailored treatments and enhance patient outcomes. The prevalent genetic changes, chromosomal abnormalities, and mutational signatures linked to laryngeal cancer are examined in this section. Many important genes involved in signal transduction, differentiation, and cell cycle regulation are frequently altered genetically in laryngeal carcinoma. With mutations seen in as many as 70% of instances of laryngeal cancer, the tumor suppressor gene TP53 is the most commonly altered gene [21,22]. TP53 mutations are linked to a poor prognosis and resistance to traditional treatments, underscoring the significance of this protein as a possible target for therapy [23]. However, genomic alterations were also detected in different cancer-relevant genes, including FAT atypical cadherin 1 (FAT1), LDL receptor-related protein 1B (LRP1B), cyclin-dependent kinase inhibitor 2A (CDKN2A), tet methylcytosine dioxygenase 2 (TET2), notch receptor 1 (NOTCH1,) and neuregulin 1 (NRG1). This results in abnormal cell cycle control and enhanced proliferation [24]. The PI3K/AKT/mTOR signaling pathway is hyperactivated when the PIK3CA gene, which codes for a catalytic subunit of phosphatidylinositol 3-kinase (PI3K), is often mutated or amplified in laryngeal cancer [25]. This route is a desirable target for therapeutic intervention since it is essential to cell survival, proliferation, and metabolism [26]. It has also been discovered that a fraction of laryngeal tumors had mutations in NOTCH1, a gene implicated in cell fate determination and differentiation, especially with loss-of-function alterations observed [27,28]. Laryngeal cancer frequently exhibits chromosomal abnormalities and copy number variations (CNVs), which lead to genomic instability and altered gene expression. Chromosomal losses are commonly seen in regions encoding tumor suppressor genes like CDKN2A (9p21) and FHIT (3p14), but recurrent gains have been seen in regions containing oncogenes like CCND1 (11q13), EGFR (7p11), and MYC (8q24) [29,30]. These chromosomal changes may result in gene deletions or amplifications, which would accelerate the growth of the tumor and affect how well it responds to treatment [31]. Complex patterns of CNVs in laryngeal cancer have been found using high-resolution genomic profiling approaches; some of these abnormalities are linked to particular clinical characteristics or outcomes. For instance, poor prognosis and lymph node metastases have been associated with amplification of the 11q13 region, which contains CCND1 and FADD [32]. In a similar vein, certain studies have linked EGFR amplification and its nuclear localization to an advanced tumor stage and a lower chance of survival [33,34]. Mutational signatures offer important insights into the genesis and possible vulnerabilities of laryngeal cancer because they reflect the underlying processes that drive genomic alterations. Due to the carcinogenic properties of polycyclic aromatic hydrocarbons in tobacco smoke, laryngeal carcinoma display a specific mutational signature linked to tobacco exposure [35]. An high tumor mutational burden (TMB) is frequently present with this profile, which could affect how well immunotherapy works [36] (Figure 1).

Additional mutational markers linked to impaired DNA mismatch repair, APOBEC enzyme activity, and age-related mutation accumulation have been identified in laryngeal cancer [37,38]. Recently, a putative signature has been proposed by analyzing a serum miRNA profile of LSCC patients for early diagnosis, including miR-93, miR-223 and miR-532 that could regulate multiple cancer-related genes [39,40]. Certain mutational signatures may be clinically significant and may influence prognosis and therapy choices. For instance, immune checkpoint drugs may have a greater effect on cancers exhibiting evidence of mismatch repair weakness [41]. The thorough analysis of the genetic landscape in laryngeal cancer has identified biomarkers and possible therapeutic targets for individualized treatment plans. Preclinical and early clinical trials on inhibitors of EGFR, cyclin-dependent kinases, and the PI3K/AKT/mTOR pathway have demonstrated promise [42]. Furthermore, the selection of patients for immunotherapy or other targeted treatments may be influenced by the discovery of particular genetic abnormalities or mutational fingerprints [43]. Translating these genetic discoveries into therapeutic practice is still difficult, though. Developing successfully targeted therapeutics is hampered by the variability of genetic modifications within and between tumors, the complexity of gene–gene interactions, and the dynamic nature of cancer evolution [44]. Moreover, a more thorough knowledge of the biology of laryngeal cancer requires the integration of genetic data with other molecular data such as epigenetic alterations and gene expression profiles.

## 4. Epigenetic Factors Influencing Laryngeal Cancer

Alongside genetic abnormalities that drive carcinogenesis, epigenetic changes are critical to the onset and spread of laryngeal cancer. Without altering the underlying DNA sequence, these reversible alterations to the genome and related proteins can have a substantial effect on gene expression. Epigenetic modifications in laryngeal carcinoma lead to the disruption of important cellular functions, such as DNA repair, apoptosis, and cell cycle regulation. The main epigenetic processes linked to laryngeal cancer are examined in this section, including non-coding RNA function, chromatin remodeling, histone changes, and DNA methylation. DNA methylation, which is the process of adding methyl groups to the cytosine residues in CpG dinucleotides, is one of the epigenetic changes in cancer that has been researched the most. Several genes, including oncogenes and tumor suppressors, exhibit abnormal DNA methylation patterns linked to laryngeal carcinoma [45]. While hypomethylation might result in enhanced gene expression or genomic instability, hypermethylation of promoter regions usually causes transcriptional silence. The hypermethylation of the O6-methylguanine-DNA methyltransferase (MGMT) promoter is one of the most clinically significant instances of DNA methylation in laryngeal cancer. By removing alkyl groups from guanine residues, the DNA repair enzyme MGMT shields cells from the mutagenic effects of alkylating chemicals. The gene is silenced when the MGMT promoter is methylated, which may make chemotherapy drugs that alkylate targets more sensitive [46,47]. Research has indicated that methylation of the MGMT promoter is linked to enhanced response to temozolomide in patients suffering from glioblastoma, and comparable studies concerning laryngeal cancer are currently in progress [48]. Other often methylated genes in laryngeal cancer include E-cadherin (CDH1), DAPK1, and p16 (CDKN2A). These tumor suppressor genes’ hypermethylation is linked to unchecked cell division, a decrease in cell adhesion, and resistance to apoptosis [49]. On the other hand, laryngeal carcinoma has been found to exhibit worldwide DNA hypomethylation, which may cause chromosomal instability and oncogene activation [50]. In cancer, chromatin remodeling and histone alterations constitute an additional layer of epigenetic control. Gene expression can be impacted by post-translational changes of histone tails, including acetylation, methylation, phosphorylation, and ubiquitination, which can change the accessibility and structure of the chromatin [51]. Changes in histone modification patterns in laryngeal carcinoma have been linked to deregulation of important pathways in the tumor’s growth. In laryngeal carcinoma, abnormal histone acetylation has been associated with the silence of tumor suppressor genes. Laryngeal cancers frequently overexpress histone deacetylases (HDACs), which remove acetyl groups from histones and encourage a closed chromatin conformation [52]. Genes implicated in cell cycle arrest and death are transcriptionally repressed as a result of this overexpression. As reported by Liu et al., HDAC inhibitors have become known as promising therapeutic drugs that have demonstrated promise in early-stage clinical trials and preclinical research for laryngeal cancer [53]. The epigenetics of laryngeal carcinoma is also significantly influenced by histone methylation. Laryngeal cancers often overexpress EZH2, a histone methyltransferase that is a part of the Polycomb repressive complex 2 (PRC2). Trimethylation of histone H3 at lysine 27 (H3K27me3) is a repressive mark linked to tumor suppressor gene silencing, which is catalyzed by EZH2 [54]. In the case of laryngeal carcinoma, targeting EZH2 and other histone methyltransferases is one possible treatment approach. Further linked to laryngeal cancer are chromatin remodeling complexes, which modify nucleosome composition and placement through ATP hydrolysis. Dysregulation of gene expression pathways involved in tumor suppression and cell differentiation may result from these changes. Non-coding RNAs have become significant epigenetic regulators in laryngeal cancer, especially microRNAs (miRNAs) and long non-coding RNAs (lncRNAs). Via many processes, including transcriptional regulation, post-transcriptional modification, and chromatin remodeling, these RNA molecules can alter the expression of genes. Laryngeal cancer has been the subject of much research on microRNAs, which are tiny non-coding RNAs that target mRNAs for translational suppression or destruction in order to control gene expression. It has been discovered that laryngeal tumors exhibit dysregulation of a wide range of miRNAs, some of which function as tumor suppressors and others as oncogenes (oncomiRs) [55]. For instance, miR-21, an oncomiR that is often overexpressed in laryngeal cancer, targets tumor suppressor genes, including PTEN and PDCD4, to encourage cell invasion and proliferation [56]. On the other hand, miR-375, which is frequently downregulated in laryngeal cancer, suppresses tumors by blocking IGF1R and increasing sensitivity to cisplatin [57]. Non-protein-coding transcripts larger than 200 nucleotides, or long non-coding RNAs, have also been linked to the etiology of laryngeal cancer. Through a variety of processes, such as chromatin remodeling, transcriptional regulation, and post-transcriptional processing, lncRNAs can control the expression of laryngeal cancer-related genes [58]. For example, the lncRNAs can promote laryngeal cancer proliferation, migration, and invasion interfering with alternative splicing processes as well as modulating miRNAs or gene expression [59,60,61]. In laryngeal cancer, the interactions between several epigenetic pathways are intricate and multidimensional. Non-coding RNAs, histone modifications, and DNA methylation frequently cooperate to control cellular processes and gene expression. For instance, promoter hypermethylation, restrictive histone marks, and the recruitment of chromatin remodeling complexes are routinely used in concert to silence tumor suppressor genes [62,63]. There are significant therapeutic ramifications for comprehending the epigenetic landscape of laryngeal carcinoma. Since epigenetic changes may be reversible, therapeutic intervention has found an appealing avenue to address them. Numerous epigenetic medications, such as HDAC inhibitors and DNA methyltransferase inhibitors, are either being studied in clinical settings or are now being used to treat a variety of malignancies, including laryngeal cancer [64]. In addition, patients with laryngeal cancer may benefit from the early identification, prognosis, and treatment response prediction provided by epigenetic biomarkers such as DNA methylation patterns or miRNA expression profiles [65,66]. Epigenetic changes are critical for the development, course, and response to treatment of laryngeal cancer.

## 5. Transcriptomic Profiling and Gene Expression Signatures

Understanding the molecular underpinnings of laryngeal cancer and its progression, response to treatment, and prognosis for patients has been made possible by the development of powerful methods such as transcriptomic profiling and the identification of gene expression signatures. Researchers have been able to thoroughly examine the transcriptome of laryngeal tumors because of high-throughput technologies like RNA sequencing and microarrays [67]. These technologies have also shown intricate patterns of gene expression that underpin the heterogeneity of the disease. In this section, the major differentially expressed genes, pathway analysis and its functional implications, and possible gene expression panels that could be used as prognostic and predictive tools for laryngeal cancer are examined. Numerous investigations comparing tumor tissues with nearby normal tissues or different tumor stages have revealed key differentially expressed genes in laryngeal carcinoma. Numerous biological processes, including cell cycle control, apoptosis, invasion, metastasis, and angiogenesis, are mediated by these genes. For example, it has been regularly reported that laryngeal cancer tissues differentially express certain genes, including EMP1, HOXB9, DPY19L2P1, MMP1, and KLHDC7B, representing independent prognosis predictor genes of laryngeal cancer [68]. A major regulator of the G1/S transition in the cell cycle, CCND1, which encodes cyclin D1, is often overexpressed in laryngeal tumors, which leads to unchecked cell proliferation [69,70]. EGFR overexpression is a significant therapeutic target since it is linked to a poor prognosis and resistance to traditional therapy [34,71,72]. On the other hand, laryngeal cancer frequently exhibits downregulation of tumor suppressor genes such as CDKN2A, PTEN, and TP53 [73]. Loss of expression for these genes can cause genomic instability, disruption of cell cycle checkpoints, and enhanced cell survival. Fascinatingly, it has been observed in certain research that laryngeal cancers exhibit paradoxical overexpression of TP53, which is frequently linked to mutant versions of the protein that lack or change tumor-suppressive properties [74]. A pathway analysis of genes with variable expression has identified several important signaling cascades that are often dysregulated in laryngeal carcinoma. The PI3K/AKT/mTOR pathway is frequently hyperactivated in laryngeal malignancies despite its critical role in cell survival and proliferation [75]. Mutations in pathway components like PIK3CA or overexpression of upstream receptors like EGFR can cause this activation in experimental models [76]. Contributing to cell survival and proliferation, the MAPK signaling pathway, which includes the RAS/RAF/MEK/ERK cascade, is another often activated route in laryngeal cancer, suggesting its role as a therapeutic target [77]. The development and metastasis of laryngeal cancer have also been linked to epithelial-to-mesenchymal transition (EMT) pathways [78]. TGF-β-Smad-mediated EMT is able to promote cell invasion and migration in the in vitro models of laryngeal cancer [79,80]. The propensity for tumor metastasis and greater tumor invasiveness is probably linked to these alterations. Different pathways have been linked to the EMT as indicated by the involvement of a variety of different proteins such as β-arrestin-1, high mobility group A2 (HMGA2), and JAK2/STAT3 signaling, adding to the characteristics of cancer uncontrolled proliferation, resistance to cell death and dysregulation of apoptosis [81,82,83]. Tumor spread is facilitated by the activation of invasion and metastasis pathways, and angiogenesis-related genes increase the blood supply to the tumor, which supports tumor growth [84].

There has been a lot of activity in the development of gene expression panels for laryngeal cancer prognostic and predictive purposes. These panels are intended to predict responsiveness to particular treatments and stratify patients according to their risk of metastasis, recurrence, or overall survival. Different profiles of the altered gene were proposed as a signature of altered genes related to the immune system, metabolism, DNA repair, and pyroptosis; these genes were linked to a poor prognosis in locally progressed laryngeal cancer, which are one noteworthy predictive gene expression signature for laryngeal cancer [85,86,87]. Although a number of encouraging gene signatures have been suggested, there are still issues with their clinical validation and use. A 7-gene signature comprising MMP1, COL4A1, and PLAU was proposed in another study by Qu et al. as a potential predictor of overall survival in patients with laryngeal cancer [88]. The use of predictive gene expression panels to inform therapy choices for laryngeal cancer has also been investigated. For example, Huang et al. provided a novel prognostic model based on six genes obtained from the Matrisome Project (FN1, LAMB4, LAMB3, DMP1, CHAD, and MMRN1) that was differentially expressed between tumor and normal samples indicating that as promising markers for clinical practice [89]. This signature contained genes related to apoptosis, cell cycle regulation, and DNA repair, underscoring the significance of these pathways in the response to treatment. With the introduction of immune checkpoint inhibitors, there has been increased interest in the possibility of immune-related gene signatures in predicting response to immunotherapy in laryngeal cancer. Studies on head and neck squamous cell carcinomas have found gene expression profiles linked to response to PD-1/PD-L1 inhibitors, albeit these findings are not unique to laryngeal cancer [90]. Genes linked to interferon-γ signaling, antigen presentation, and T-cell activity are frequently included in these signatures. The practical application of these gene expression panels still faces several obstacles, notwithstanding their potential. The use of gene signatures obtained from bulk tumor investigations may be complicated by the heterogeneity of laryngeal cancers, both intra- and intertumoral. Furthermore, these signatures’ validity and replication across various patient populations and technological platforms continue to be crucial challenges [91]. Transcriptomic profiling in laryngeal cancer has new opportunities thanks to recent developments in single-cell RNA sequencing methods. These methods make it possible to characterize the patterns of gene expression in individual cells, offering insights into the microenvironment of the tumor, heterogeneity, and unusual cell populations that might be important for the development of the disease or resistance to treatment [92]. Different subpopulations of infiltrating cells in the laryngeal tumor microenvironment have been identified by single-cell transcriptomics, highlighting complex and specific cell signatures that play a critical role in tumor progression and metastasis formation [93]. These subpopulations may be responsible for treatment resistance and recurrence of the disease. More and more researchers are realizing how important it is to combine transcriptome data with other molecular profiling techniques like proteomics, genomics, and epigenomics to fully comprehend the biology of laryngeal cancer. Multi-omics investigations can uncover more reliable biomarkers and therapeutic targets as well as intricate relationships across several molecular layers [94].

## 6. Biomarkers of Tumor Microenvironment and Immune Response

**Figure 2:**
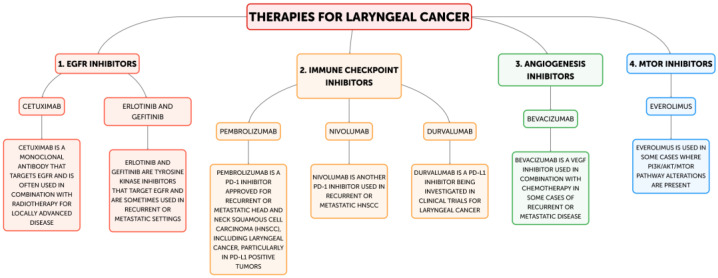
Personalized and targeted therapies available.

Tumor microenvironment (TME): Development, progression, and response to treatment of laryngeal cancer are all significantly influenced by TME. The significance of comprehending the intricate relationships between tumor cells and the immune system has been brought to light by recent developments in cancer immunology [95]. This section focuses on tumor mutational burden (TMB), immunological checkpoint molecules, tumor-infiltrating lymphocytes (TILs), and microsatellite instability (MSI) as important indicators of the TME and immune response in laryngeal cancer. Immune checkpoint molecules have become significant diagnostic and therapeutic targets in laryngeal cancer, with PD-L1 (Programmed Death-Ligand 1) and CTLA-4 (Cytotoxic T-Lymphocyte Associated Protein 4) being the most prominent examples. Cancer cells can avoid immune surveillance when PD-L1 expression is present on tumor cells or immune cells in the tumor microenvironment (TME) (Figure 2).

This can decrease T-cell responses. PD-L1 expression in laryngeal cancer has been the subject of numerous investigations, with variable degrees of success based on the detection techniques and cut-off values employed [96]. Wang et al.’s meta-analysis revealed that head and neck squamous cell carcinoma (HNSCC), which includes laryngeal cancer, had a low overall survival rate when PD-L1 expression was present [97]. The predictive significance of PD-L1 expression is still debatable, nevertheless, as certain research indicates that there is no meaningful correlation with survival rates [98]. Its utility as a stand-alone biomarker is complicated by the variability of PD-L1 expression within tumors and the dynamic nature of its regulation. Compared to PD-L1, CTLA-4, another immune checkpoint molecule, has not been as thoroughly researched in laryngeal cancer cases. T-cells express CTLA-4, which inhibits T-cell activation by interacting with antigen-presenting cells’ CD80/CD86. Although CTLA-4 inhibition has demonstrated potential in other forms of cancer, its function as a biomarker for laryngeal cancer is still unclear [43]. Tumor-infiltrating lymphocytes (TILs) have been identified as significant prognostic indicators for several malignancies, including laryngeal carcinoma, based on their composition and existence. T lymphocytes (TILs) are the host immune response against the tumor. They are divided into many subsets, such as NK cells, CD4+ helper T cells, CD8+ cytotoxic T cells, and regulatory T cells (Tregs). TIL density, phenotype, and location can reveal important details regarding the tumor’s immune state and possible responsiveness to immunotherapy and attribute to them a prognostic significance in laryngeal carcinoma [99]. Nguyen et al. discovered that patients with laryngeal squamous cell carcinoma had better overall survival rates when there were higher numbers of CD8+ T lymphocytes in the tumor epithelium [100]. Recently, Xirou et al. indicated that TIL variables evaluated through a machine-based method could be considered as independent predictors of disease-free survival in laryngeal cancer [101]. Research has also looked into the function of various T-cell subsets in the prognosis of laryngeal cancer [102]. While having a lot of CD8+ T cells is usually associated with better results, some studies have connected Tregs, which are identified by the expression of FOXP3, to a worse prognosis [103]. The balance of immunosuppressive cells and effector T cells within the TME seems to have a crucial role in dictating treatment results and disease outcomes. A promising biomarker for the response to immunotherapy in several cancer types, including laryngeal carcinoma, is tumor mutational burden (TMB). The total number of somatic mutations per coding region of the tumor genome is known as TMB. Elevated TMB is assumed to be correlated with more neoantigens, which could result in greater immune system detection and response to immune checkpoint inhibitors [104]. The relevance of TMB in laryngeal cancer is currently being studied, even though it has demonstrated potential as a predictive biomarker for immunotherapy response in certain cancer forms. High TMB was linked to better overall survival in HNSCC patients receiving anti-PD-1/PD-L1 therapy, according to research by Hanna et al. [105]. Research is still ongoing to determine the best cut-off values for identifying high TMB in laryngeal cancer and how to combine it with other biomarkers. A further possible biomarker associated with the genetic landscape of the tumor is microsatellite instability (MSI). Mutations accumulate in repeating DNA sequences called microsatellites (MSI) as a result of flaws in the DNA mismatch repair (MMR) machinery. Improved response to immune checkpoint drugs has been linked to high levels of MSI (MSI-H) in a variety of cancer types [41]. Compared to several other cancer forms, the frequency of MSI-H tumors in laryngeal cancer is rather low. Just 3% of HNSCC patients, including laryngeal malignancies, had MSI-H status, according to research by Hayashi et al. [81]. Even though MSI testing is uncommon, it might still be important for some patients with laryngeal cancer, especially when it comes to customized immunotherapy strategies. Combining several biomarkers, such as TILs, TMB, MSI, and PD-L1 expression, may allow for a more thorough evaluation of the immune environment in laryngeal cancer. Combination biomarker techniques are being investigated for laryngeal cancer since they have demonstrated potential in other cancer types. For instance, PD-L1 expression and TMB together may be able to more accurately predict the response to pembrolizumab in a variety of solid tumors, including HNSCC, according to a study by Cristescu et al. [106]. Advances in spatial transcriptomics and single-cell technology have made it possible to characterize the TME in laryngeal carcinoma in greater depth. By identifying uncommon cell types and figuring out how immune cells are arranged inside tumors, these methods can shed light on immune evasion strategies and possible treatment targets [107]. There is increasing interest in the role of other TME constituents, such as tumor-associated macrophages (TAMs) and cancer-associated fibroblasts (CAFs), in the development of laryngeal cancer and its response to treatment. CAFs promote cell proliferation, colony formation, EMT, and tumorigenesis in laryngeal squamous cell carcinoma (LSCC) via extracellular vesicle production [108]. These stromal cells may function as extra indicators or therapeutic targets in addition to contributing to an immunosuppressive environment [109]. The biomarkers of the immune system and the tumor microenvironment provide important information about the biology of laryngeal cancer and may influence treatment choices, especially in the age of immunotherapy [108]. Even while certain biomarkers, such as PD-L1 expression, TILs, TMB, and MSI, have demonstrated potential, it is still difficult to include them in all-encompassing prediction models. Standardizing biomarker evaluation techniques, investigating new biomarkers, and creating multi-parameter predictive models ought to be the main areas of future study to maximize patient selection for immunotherapy and other targeted treatments for laryngeal cancer.

## 7. Cancer Stem Cell Markers in Laryngeal Cancer

According to the theory of cancer stem cells (CSCs), a tiny percentage of tumor cells have characteristics similar to those of stem cells, such as the ability to self-renew and differentiate. It is believed that these CSCs are essential for the development, spread, metastasis, and resistance to treatment of many malignancies, including laryngeal carcinoma [110]. Research on the identification and characterization of CSCs in laryngeal cancer has gained significant traction due to its potential impact on treatment approaches, prognosis, and diagnosis. Among the most extensively researched CSC markers in laryngeal cancer are CD44 and the aldehyde dehydrogenase 1 family member A1 (ALDH1A1). A glycoprotein found on the cell surface, CD44, is involved in migration, adhesion, and interactions between cells. Numerous biological activities, including tumor growth and metastasis, have been linked to it [111]. The expression of CD44 in laryngeal carcinoma and its clinical importance have been the subject of numerous investigations. Laryngeal cancer tissues are characterized by a considerable increase in CD44 expression when compared to nearby normal tissues [112]. High levels of CD44 expression were linked to lymph node metastases, poor differentiation, and an advanced and more aggressive clinical stage of primary tumors [113,114]. Increased expression of CD44 was indicated as an independent predictor of a poor prognosis in early-stage laryngeal cancer [113]. An intracellular enzyme called ALDH1A1 is involved in the oxidation of aldehydes and is a hallmark of CSCs in several different forms of cancer. ALDH1A1 expression has been linked to CSC characteristics and unfavorable clinical outcomes in laryngeal cancer. ALDH1A1-positive cells derived from laryngeal cancer cell lines showed increased tumorigenic capacity, resistance to chemotherapy, and self-renewal, as Wu et al. demonstrated [115]. It has been suggested that the co-expression of ALDH1A1 and CD44 is a more precise marker for locating CSCs in laryngeal cancer. Compared to CD44-/ALDH1A1- cells, Qi et al. discovered that CD44+/ALDH1A1+ cells isolated from laryngeal carcinoma tissues displayed higher tumor-initiating capacity and resistance to cisplatin [116]. Furthermore, patients with laryngeal cancer had a poorer prognosis when CD44+/ALDH1A1+ cells were present. Several other potential CSC markers have been studied in laryngeal cancer, in addition to CD44 and ALDH1A1. Among these is CD133, sometimes referred to as prominin-1; CD133 is a CSC marker in several solid cancers. In laryngeal carcinoma, CD133 expression was linked to poor differentiation, an advanced clinical stage, and lymph node metastases [117]. In vitro and in vivo, CD133-positive cells isolated from laryngeal cancer tissues showed a higher propensity for self-renewal and tumorigenesis [118,119]. Sox2, a transcription factor crucial in preserving stem cell characteristics, has been connected to the laryngeal carcinoma CSC phenotype. Sox2 plays an important role in the early stage of tumorigenesis in laryngeal cancer tissues when compared to normal laryngeal tissues [120]. Additionally, high Sox2 expression was linked to lymph node metastases, poor differentiation, and an advanced clinical stage [121,122]. Another transcription factor associated with stemness, Oct4, has also been investigated in CSCs from laryngeal cancer. In laryngeal cancer tissues, Cao et al. showed a positive correlation between Oct4 expression and CD44 expression. Moreover, high Oct4 expression was linked to a poor prognosis [93]. BMI1, a protein belonging to the polycomb group, has been connected to CSC self-renewal. BMI1 expression was discovered to be elevated in laryngeal cancer tissues and cell lines [123]. BMI1 knockdown decreased the proliferation, invasion, and stem cell-like characteristics of laryngeal cancer cells. Another member of the ATP-binding cassette (ABC) transporter family, ABCG2, has also been linked to drug resistance in some cancer types as well as the CSC phenotype. Recently, a positive correlation between poor differentiation, an advanced clinical stage, chemoresistance, and higher expression of ABCG2 was highlighted in laryngeal cancer tissues [124,125]. These CSC markers have important clinical consequences for laryngeal cancer. Numerous clinicopathological characteristics, including tumor grade, stage, metastasis, and patient prognosis, have been linked to their expression. Furthermore, it has been shown that the existence of CSCs is associated with resistance to treatment, specifically to radiation and conventional chemotherapy [112,126]. The discovery of CSC markers in laryngeal cancer has created new treatment opportunities. Targeting CSCs has been approached in several ways. Potential therapeutic drugs have been studied, which target CSC surface markers, such as CD44 or CD133, with antibodies or other compounds. A liposomal nanoparticle delivery method that targets CD44 can efficiently target and eradicate CD44-positive laryngeal cancer cells, as shown by Nozaki et al. [127]. CSC maintenance and self-renewal have been linked to inhibiting Wnt, Notch, and Hedgehog signaling pathways. Preclinical research has demonstrated the promise of inhibitors that target these pathways. Notch expression was shown to be associated with low survival in moderate/ poor differentiated human oral squamous cell carcinoma patients, indicating its potential as a biomarker [128]. Histone deacetylase (HDAC) inhibitors are one example of an epigenetic regulator that has been demonstrated to target CSCs in a variety of malignancies. HDAC inhibitor has been shown to inhibit the CSC stemness by reducing markers NANOG and Survivin expression and, in turn, increasing the sensitivity of laryngeal cancer cells to dihydropyrimidine dehydeogenase (DDP)-based chemotherapy [129]. A further possible therapeutic strategy is to induce the differentiation of CSCs into more mature, less tumorigenic cells. It has been demonstrated that the vitamin A derivative retinoic acid can cause differentiation and decrease the number of CSCs in laryngeal cancer cells [100]. Research on using immunotherapy techniques to target CSCs, such as cancer vaccines or chimeric antigen receptor (CAR) T-cell treatment, is only being started. These strategies have demonstrated promise in other cancer types and may be applicable to laryngeal CSCs, despite not having been particularly examined in laryngeal cancer [130]. Enhancing results and overcoming treatment resistance may be possible by combining CSC-targeted medicines with traditional treatments. Xu et al. showed that by targeting both CSCs and non-CSCs, the combination of the ALDH inhibitor disulfiram with cisplatin increased the efficacy of chemotherapy in laryngeal cancer cells [102]. Although preclinical results are encouraging, there are still a number of obstacles to overcome before CSC-targeted medicines can be used in patients. These include the need for more precise CSC markers to reduce off-target effects on normal stem cells, the variability of CSC populations, and the possibility of CSC plasticity [131].

## 8. Integration of Molecular Data with Clinicopathological Factors

A major development in the treatment of laryngeal cancer is the incorporation of molecular data with conventional clinicopathological variables. This method seeks to enhance prognostic precision, offer a more thorough grasp of tumor biology, and direct individualized treatment plans. More advanced tools for patient stratification and decision-making can be created by academics and clinicians by combining different biomarkers and clinical characteristics. The creation of integrated prognostic models for laryngeal cancer that incorporate clinicopathological variables and molecular biomarkers has been the subject of several investigations. The goal of these models is to increase the precision of therapy response evaluation and prognosis prediction. In one such study, Zhang et al. created a nomogram to predict overall survival in patients with laryngeal squamous cell carcinoma [132]. The nomogram combined clinical criteria, such as TNM stage and tumor differentiation, with genetic markers, such as p53, EGFR, and Ki-67 expression. The possibility of combining molecular data with standard staging techniques was highlighted by the combined model’s better prediction performance when compared to TNM staging alone. In a different investigation, Wildeman et al. developed a predictive model to predict locoregional control in laryngeal cancer patients receiving radiation therapy by combining clinical variables (T-stage, N-stage, and tumor site) with genetic markers (p53 and EGFR expression) [133]. When compared to models that were only based on clinical criteria or molecular markers, the integrated model showed better prediction accuracy. New developments in high-throughput transcriptomic and genomic technologies have made it possible to create prognostic models with more complexity. For example, Chen et al. used gene expression profiling to find a 15-gene signature that greatly increased predictive accuracy in patients with laryngeal cancer when paired with clinical variables [134]. Significant insights into tumor biology and possible treatment targets were obtained by this comprehensive approach. Better risk classification techniques for patients with laryngeal cancer have been developed as a result of the integration of molecular data with clinicopathological variables. By identifying patient subgroups with unique prognostic profiles and treatment response patterns, these initiatives hope to provide more individualized approaches to patient care. A noteworthy instance is the work of Foy et al., who created a risk stratification system for patients with laryngeal and hypopharyngeal tumors that included both molecular markers (p16 and EGFR expression) and clinical criteria (T-stage, N-stage, and extracapsular dissemination) [135]. With three discrete risk groups and noticeably differing survival results, this approach offered a useful tool for patient counseling and treatment planning. In a different study, Mes et al. suggested a method for risk stratification that divided laryngeal cancer patients into low-, intermediate-, and high-risk groups based on a panel of genetic changes (TP53, CDKN2A, and CCND1), clinical variables, and HPV status [18]. When compared to traditional staging approaches, this combined strategy showed greater predictive power and indicated the potential of de-escalating treatment in low-risk individuals. Recently, there has been increased interest in the use of immune-related biomarkers in risk classification techniques. An immune-related predictive model, for instance, was created by Zhang et al., that incorporated immunological checkpoint molecule expression, immune cell infiltration patterns, and clinical variables [136]. In addition to increasing prognostic accuracy, this integrated method offered insights into possible immunotherapy approaches for various patient subgroups. Treatment decisions for laryngeal cancer are significantly impacted by the integration of molecular data and clinicopathological variables. These integrated techniques offer a more thorough understanding of tumor biology and patient-specific risk factors, which can help pick the best course of treatment and identify patients who can benefit from new targeted treatments or immunotherapies. Integrated techniques have demonstrated potential in directing organ preservation strategies. For example, Dietz et al. created a decision support system to predict the chance of effective larynx preservation in patients receiving induction chemotherapy by combining clinical variables, molecular markers (EGFR, p53), and functional imaging data [137]. By using an integrated strategy, it was possible to identify individuals who would benefit most from organ preservation regimens and avoid needless, drastic surgery. Integrated prognostic models have been applied to radiation therapy to direct treatment de-escalation or intensification. According to research by Senghore et al., clinical outcomes in patients with laryngeal cancer could be predicted using a combination model that included clinical characteristics and the polymorphisms of mismatched DNA repair genes [138]. This method might be used to determine which patients would benefit from adding radiosensitizing drugs or increasing the dose. The identification of patients who could benefit from targeted therapy has also been made possible by the integration of molecular data. For instance, Bossi et al. created a biomarker-based algorithm to direct the use of EGFR inhibitors in head and neck tumors, including laryngeal carcinoma [139]. This algorithm integrated EGFR expression, gene copy number, and mutation status. With the use of an integrated approach, patient selection for targeted medicines was improved, perhaps leading to better treatment outcomes and a decrease in needless toxicities. Integrated techniques have grown in significance for patient selection in the immunotherapy era. To guide the use of immune checkpoint inhibitors in head and neck tumors, Mandal et al. established a framework that included tumor mutational burden, immune cell infiltration patterns, and PD-L1 expression [140]. The integrated method was designed to overcome the drawbacks of single-biomarker techniques by identifying patients who are most likely to react to immunotherapy. Although integrated techniques have shown encouraging outcomes, there are still a number of obstacles in the way of their general clinical deployment. Molecular testing procedures need to be standardized, prognostic models need to be validated in large, heterogeneous patient cohorts, and user-friendly tools for clinical decision-making need to be developed [31]. Moreover, the dynamic character of tumor biology and the evolution of mechanisms for resistance to therapy emphasize the necessity of adaptive tactics that can take into account long-term molecular data. Liquid biopsies and circulating tumor DNA (ctDNA) analysis are two promising approaches for real-time molecular change and therapy response monitoring [141].

## 9. Challenges and Future Directions

Although there is a lot of potential in the integration of molecular data into the clinical therapy of laryngeal cancer, there are still several issues that need to be resolved. Researchers and physicians must overcome these challenges as the area develops while investigating novel technology and methods to enhance patient outcomes. The present obstacles, new developments, and potential applications of personalized medicine in laryngeal cancer are covered in this section. A major obstacle in the application of molecular testing for laryngeal cancer is the absence of uniformity among various laboratories and establishments. This unpredictability may ultimately impede the general adoption of molecular-based techniques in clinical practice by producing inconsistent results and making it difficult to compare data across investigations. Several professional associations have started creating standards for molecular testing in head and neck cancers, including laryngeal carcinoma, to solve this problem. For instance, guidelines for HPV testing in head and neck carcinomas have been jointly published by the American Society of Clinical Oncology (ASCO) and the College of American Pathologists (CAP) [142]. For testing procedures and other molecular markers, comparable standardized techniques are required. Several important areas should be the focus of efforts to standardize molecular testing. It is imperative to establish standard operating procedures for tissue collection, preservation, and processing to guarantee reliable and superior-quality samples for molecular analysis. Inter-laboratory variability can be decreased by standardizing the methods used for molecular testing, such as next-generation sequencing, in situ hybridization, and immunohistochemistry. Consensus criteria for the interpretation and reporting of molecular test results should be developed to promote uniformity and ease clinical decision-making. Maintaining high standards across laboratories can be facilitated by putting in place strict quality control procedures and frequent proficiency testing programs. Our knowledge of the molecular landscape of laryngeal cancer can be enhanced, and research collaborations facilitated by the establishment of centralized databases for molecular data [143]. Academic institutions, professional associations, and regulatory bodies must work together to address these standardization difficulties. To expedite progress in this area, multinational consortia and working groups devoted to standardizing molecular testing in laryngeal cancer might be formed. Technological developments in molecular profiling are contributing to our ongoing capacity to better understand the proteomic, transcriptomic, and genomic landscapes of laryngeal cancer. Better prognostic and predictive biomarkers may result from the more thorough and accurate molecular characterization that these developing technologies provide. Through the examination of individual cells within a tumor, single-cell sequencing can reveal intratumoral heterogeneity and identify uncommon cell types like cancer stem cells. Head and neck malignancies have already been treated with single-cell RNA sequencing, which has identified unique molecular subgroups and possible treatment targets [92]. Gene expression analysis and spatial data are combined in spatial transcriptomics, enabling researchers to map the distribution of various cell types and genetic markers inside the tumor microenvironment. Regarding immune cell infiltration patterns and tumor–stroma interactions in laryngeal cancer, this technology may offer important new insights [144]. Liquid biopsies provide a minimally invasive way to evaluate therapy response and perform molecular profiling by analyzing circulating tumor DNA (ctDNA) and circulating tumor cells (CTCs) in blood samples. Liquid biopsies have shown promise in recent times for early identification, prognostication, and therapy response monitoring in head and neck cancers, including laryngeal carcinoma [145]. A more thorough examination of the protein and metabolite profiles in laryngeal cancer is now possible thanks to sophisticated mass spectrometry techniques. The identification of novel biomarkers and therapeutic targets may result from these proteomics and metabolomics techniques [146]. Prognostic models and treatment decision support systems may be enhanced by the combination of clinical data, molecular data, and machine learning techniques. These tools have demonstrated promise in the analysis of intricate datasets, revealing patterns that conventional statistical techniques would miss [147]. The thorough validation and standardization of these technologies will be necessary for their inclusion into clinical practice as they continue to evolve. Additionally, to help doctors translate molecular results into practical treatment recommendations, the interpretation of complicated molecular data will require the creation of user-friendly tools and decision support systems. Ultimately, molecular profiling in laryngeal cancer aims to provide genuinely customized treatment plans that optimize effectiveness while reducing side effects. There are several exciting new directions in targeted medicines and customized medicine. Research on optimizing immunotherapy is ongoing because immune checkpoint inhibitors have been demonstrated to be effective in certain laryngeal cancer patients, although response rates are still not at their best. In order to improve the outcomes of immunotherapy, research is still being conducted to uncover predictive biomarkers and combination tactics. For example, immune checkpoint inhibitors can be used in conjunction with targeted medicines or radiation therapy to boost anti-tumor immune responses [43]. The growing understanding of the molecular causes of laryngeal cancer is opening up new avenues for the development of sensible medication combinations. For example, preclinical research and early-phase clinical trials have demonstrated the potential benefits of combining autophagy inhibition with drugs that target downstream signaling pathways, such as PI3K/AKT/mTOR inhibitors [148]. Innovative drug delivery strategies, like hydrogels and nanoparticle-based methods, are being created to boost the effectiveness of targeted treatments for head and neck tumors while lowering their toxicity. These methods may reduce systemic adverse effects while facilitating more targeted medication delivery to the tumor site [149]. The creation of real-time monitoring methods, such as liquid biopsies, may make adaptive treatment plans possible, which modify therapy in response to changes in the patient’s molecular makeup. By using this strategy, treatment resistance may be overcome, and long-term results may be enhanced [150]. By combining molecular profiling with cutting-edge radiation methods like proton treatment or MRI-guided radiotherapy, tumor tissue may be more precisely targeted while protecting healthy tissues. The application of radiosensitivity gene signatures to direct de-escalation or escalation techniques for radiation exposure is now being investigated [151]. Novel therapeutics targeting cancer stem cells in laryngeal carcinoma are being explored as our understanding of these cells expands. Targeting particular surface markers or signaling pathways related to stem cell maintenance are examples of strategies that may enhance treatment results and lower recurrence rates [152]. There is growing evidence linking epigenetic modifications to laryngeal carcinoma. Histone deacetylase inhibitors and DNA methyltransferase inhibitors are examples of epigenetic modifiers that are being studied as possible therapeutic possibilities, either on their own or in conjunction with other therapies [153]. Although these strategies have a lot of potential, a few obstacles need to be overcome before customized treatment for laryngeal cancer can reach its full potential. Personalized therapy techniques might not be a good fit for conventional clinical trial designs. To effectively assess targeted medicines, new adaptive trial designs and basket trials based on molecular profiles rather than tumor location classifications may be required [154]. Cost and accessibility concerns need to be addressed as molecular diagnostics, and targeted medicines grow more sophisticated to guarantee that all patients have fair access to individualized treatment plans. Clinicians must receive continual education and training in order to comprehend complicated molecular data and apply personalized medicine techniques. The use of whole genome profiling presents moral dilemmas for the handling of germline mutations and coincidental discoveries that could affect the relatives of patients.

## 10. Conclusions

The use of gene expression biomarkers for molecular profiling of laryngeal cancer has greatly advanced our knowledge of and ability to treat this difficult illness. The present understanding of significant genetic mutations, epigenetic modifications, and gene expression profiles that impact the pathophysiology of laryngeal cancer and its prognosis has been compiled in this review. Personalized treatment planning and improved prognostication are possible when these molecular insights are combined with conventional clinicopathological variables. Still, there are several obstacles to overcome before these results may be applied in standard clinical practice. Three main difficulties need to be addressed: the cost-effectiveness of thorough profiling, the standardization of molecular testing techniques, and the interpretation of complicated genetic data. Furthermore, to confirm the clinical usefulness of gene expression profiles and molecularly guided therapeutic strategies, prospective clinical trials are required. The field of tailored treatment for laryngeal cancer is developing quickly despite these obstacles. Future developments in single-cell sequencing and liquid biopsies will probably improve our capacity to track therapy response and define tumor heterogeneity. Treatment plans that are more efficient, less hazardous, and genuinely customized are possible as our knowledge of the molecular landscape of laryngeal cancer advances. Even though there has been a lot of progress, more study and cooperation between physicians, molecular biologists, and bioinformaticians are necessary to attain the full potential of personalized treatment in laryngeal cancer.

## Data Availability

No new data were created, or where data is unavailable due to privacy or ethical restrictions, a statement is still required.
